# Activity interference in patients with Sjögren’s syndrome: a cross-sectional study of 149 patients in the UK

**DOI:** 10.1093/rheumatology/keac053

**Published:** 2022-01-30

**Authors:** Hannah Schoon, Emma Slack, Mark Pearce, Wan-Fai Ng, Katie L Hackett

**Affiliations:** Southeast Utah Health Department, Price, UT, USA; Faculty of Medical Sciences; Population Health Sciences Institute; Population Health Sciences Institute; NIHR Newcastle Biomedical Research Centre, Newcastle University; Department of Social Work, Education and Community Wellbeing, Northumbria University, Newcastle upon Tyne, UK

**Keywords:** Sjögren’s syndrome, activity interference, autoimmunity

## Abstract

**Objectives:**

To investigate which five activity interference categories out of pain, fatigue, mood, dryness and brain fog/mental fatigue scored highest in patients with primary Sjögren’s syndrome (pSS) and to investigate the association between activity interference and mood and physical functioning in these patients.

**Methods:**

The Comprehensive Pain Evaluation Questionnaire (CPEQ) assessed activity interference (actions performed in daily life that are hindered) in 149 UK pSS patients. This was modified to include four additional symptoms (fatigue, mood, dryness and brainfog/mental fatigue). Functional impairment was measured using the Hospital Anxiety and Depression Scale (HADS) and the Improved Health Assessment Questionnaire (Improved HAQ). Univariable linear regression models were estimated to investigate the association between CPEQ results and the outcome scores obtained from the HADS and Improved HAQ. Multivariable linear regression models were estimated adjusting for patient age and length of disease.

**Results:**

Fatigue had the biggest impact on seven activity domains: physical exercise (mean score of 3.49 out of 5 [s.d. 1.26]), performing household chores (mean 3.14 [s.d. 1.18]), gardening or shopping (mean 3.18 [s.d. 1.20]), socializing with others (mean 2.62 [s.d. 1.24]), recreation/hobbies (mean 2.88 [s.d. 1.20]), sexual relations (mean 3.00 [s.d. 1.52]), and mental efficacy (mean 2.69 [s.d. 1.17]). Regression analysis showed a positive correlation in which every point increase in an activity interference category saw the overall mood and physical functioning scores increase.

**Conclusion:**

Fatigue has the largest impact on pSS patients’ daily activities in this cohort. Length of disease reduced the impact of activity interference on patients’ overall health score.

Rheumatology key messages

- In this group of pSS patients, fatigue had the largest impact on activity interference.
- Longer duration of a clinical diagnosis resulted in better quality of life in pSS patients.
- Activity interferences experienced with pSS are significantly interconnected with one another.

## Introduction

Primary Sjögren’s syndrome (pSS) is classified as the second most common autoimmune disease in the world, closely behind RA, with a variable prevalence rate of 0.5–2.0% [1–3]. Incidence is just as varied, with estimates ranging between 3.9 and 6.0 per 100 000 people [1, 3, 4]. Like most autoimmune diseases, it is more common in women, with evidence showing a 20:1 female to male ratio [4]. Those with pSS disease are classified by defective lacrimal (eye) and salivary glands, along with exocrine gland inflammation [5]. pSS is a complex disease that is still not completely understood [4]. Research has consistently reported an increased risk of acquiring secondary diseases (non-Hodgkin B cell lymphoma (risk ratio of 13.76; 95% CI: 8.53, 18.9 [6]), lung disease (up to 75% of pSS patients [7]), and myocardial infarctions (*P*-value 0.00 2 [8]) after a pSS diagnosis, causing further complications [6–8]. Oral and eye health are universal complaints made by both male and female patients [5]. Keratoconjunctivitis sicca that leads to xerophthalmia (dry eyes disease) is the most commonly reported eye condition with 95% of patients reporting symptoms [5, 9].

Due to pSS’s chronic and integral pathology, patients’ quality of life is significantly affected through multiple different avenues. Numerous studies have used self-reported questionnaires to look at the general quality of life affected by pSS [2, 10–12]. One study conducted in the USA found that patients with pSS are more likely to be hospitalized, experience infections and require the use of multiple medications [10]. Out-of-pocket spending on dental care has also been shown to be 3-fold higher in those with pSS in the USA [11]. Those in the UK are not sheltered from similar socio-economic effects, with patients spending between £9800 and £15 700 annually on direct (defined as ‘the value or resources used in the diagnosis, treatment and rehabilitation of a disease’) and indirect (‘economic productivity lost due to the disease’) healthcare costs [12, 13]. In 2005, the US Sjögren’s Syndrome Foundation surveyed over 3000 patients and found the average time to diagnosis after the initial presentation of symptoms took over 6 years [5]. Qualitative evidence suggested that this delay in diagnosis increased mental and family struggles for patients with pSS [14, 15].

Compared with other autoimmune diseases, research into pSS is not as advanced, and further exploration into activity interference among these patients is even less so. Previous research has only looked at one symptom or else taken a broader approach and analysed only the quality of life [2, 10–12]. This study goes further by looking at multiple different pSS symptoms to observe possible activity interferences that patients may face in their daily lives. We have also evaluated mental health symptoms, using the Hospital Anxiety and Depression Scale (HADS), physical constraints and an overall health assessment, an analysis that to our knowledge has not previously been explored in depth. By looking at several aspects of patients’ symptoms, including mood, dryness, fatigue, pain and brain fog/mental fatigue, we can obtain a clearer insight into pSS patients and their daily struggles. Including HADS allows us to investigate mental health symptoms experienced by Sjögren’s patients. Due to the unclear pathology of pSS, treatment is centred on symptoms rather than the disease itself. Further knowledge of how these symptoms interfere with a patient’s activity level could shift the research focus onto treatments that are effective for the most limiting symptoms.

## Methods

This study uses anonymized, cross-sectional quantitative data on patients with pSS (*n* = 149) from a previous mixed methods study engaging PSS patients, family members and healthcare professionals to identify barriers and facilitators to participation in life activities [16]. Data collection methods are described in detail elsewhere [17]. In summary, patients who were diagnosed using the American European Consensus Group (AECG) classification were recruited to participate in the UK Sjögren’s Syndrome Registry (UKPSSR) [16–18]. This database consists of pSS patients from different areas in the UK who have consented to be contacted for future research [16–18]. Using the UKPSSR, patient enrolment occurred between February and August of 2014 from 12 locations throughout England [16]. An invitation packet was mailed out with pSS formulated questionnaires [16]. Regulations outlined in the Helsinki Declaration were used to obtain consent from all participants [16, 19].

A favourable ethical opinion for the original study was obtained from the Office for Research Ethics Committee of Northern Ireland (13/NI/0190), and the committee specifically approved this study. Participants provided written informed consent prior to taking part. The anonymized dataset is available on request to the corresponding author.

### Exposure: activity interference assessment questionnaires

The Comprehensive Pain Evaluation Questionnaire (CPEQ) was developed to understand both physical and psychological symptoms of the chronic pain patients suffer from [20]. Only portion A, the activity interference portion, of the CPEQ was used in this analysis [20]. The original CPEQ asks ‘During the past month, how much did *pain* interfere with the following activities?’ [20] Activities such as yard work, sexual relations and going to work were included in the questionnaire [20]. Participants were asked to complete the interference portion four further times and the main word *pain* was replaced with four additional symptoms [16]: fatigue, mood, dryness and brain fog/mental fatigue, e.g. ‘During the past month, how much did *mood* interfere with the following activities?’ [16, 20] A total of five different versions of the CPEQ were administered to understand and compare the different activity interferences that pSS patients face. Each CPEQ was scored using a 5-point scale where 1 = ‘not at all’, 2 = ‘a little bit’, 3 = ‘moderately’, 4 = ‘quite a bit’ and 5 = ‘extremely’ [20]. Higher scores indicated a greater lifestyle impact. These questionnaires will be referred to as the Pain Activity Interference Questionnaire, Fatigue Activity Interference Questionnaire, Mood Activity Interference Questionnaire, Dryness Activity Interference Questionnaire and Brain Fog/Mental Fatigue Activity Interference Questionnaire for the remainder of this paper. These additional symptoms were chosen as our previous work has suggested that these symptoms interfere with the ability to perform daily activities in pSS patients [18].

### Outcome: Hospital Anxiety and Depression Scale

The Hospital Anxiety and Depression Scale (HADS) was developed in 1983 by Snaith and Zigmond [16, 21]. This self-assessment questionnaire has been used to understand and classify states of anxiety and depression in patients [21]. The questionnaire is split in half with seven questions concerning anxiety and seven on depression. Questions are scored on a 4-point scale where 0 = ‘not at all’, 1 = ‘from time to time/occasionally’, 2 = ‘a lot of the time’ and 3 = ‘most of the time’. The final score is between 0 and 21 with a higher score correlating to a worse mental prognosis . A score of >10 on either subscale indicates a case for anxiety or depression.

### Outcome: Improved Health Assessment Questionnaire

The Improved Health Assessment Questionnaire (Improved HAQ) is used to measure physical function unlike HADS, which measures the mental health symptoms of anxiety and depression [22]. There are a total of 24 questions that comprise the Improved HAQ, 20 questions that ask about the patient’s activity level and four that ask about the use of help [22]. The 20 activity questions rate a patient’s ability on a 5-point scale with 0 = ‘without any difficulty’, 1 = ‘with a little difficulty’, 2 = ‘with some difficulty’, 3 = ‘with much difficulty’ and 4 = ‘unable to do’ [16, 22]. The 20 questions that ask about ability are divided into eight different physical functioning domains (eating, walking, arising, dressing, grip, hygiene, reach and activity) [23]. The question that scores the highest in each domain is used as the domain score [23]. The total Improved HAQ score is calculated by the sum of all eight domain scores, divided by 8 then multiplied by 25 [23]. The final score is between 0 and 100 and a higher total score relates to a greater functional impairment [16, 22]. Data used in this research are based on the total Improved HAQ score and not the four questions concerning the use of patient help [16].

### Additional variables

Information was also available on self-reported patient characteristics. Categorical variables included: employment status, with coding 1–6 (1: unemployed, 2: part time, 3: full time, 4: full time education, 5: housewife/husband, 6: retired); other adults living in the household (1: yes, 2: no); dependents (1: yes, 2: no); paid for non-NHS services (1: yes, 2: no); qualifications (1: no formal qualifications, 2: GCSE/O level, 3: A level, 4: university degree, 5: postgraduate degree); household income (1: ≤£15 000, 2: £15 001–£26 000, 3: £26 001–£35 000, 4: £35 001–£50 000, 5: £5000–£70 000, 6: ≥£70 001); disability allowance (Disability Living Allowance /Personal Independence Payments) (1: yes, 2: no); and either Employment and Support Allowance or Incapacity Benefit (1: yes, 2: no). Continuous variables of age and length of disease and binary variable gender (1: female, 2: male) were included. All variables were recorded using methods discussed elsewhere [17].

### Statistical methods/analysis

Descriptive statistics were calculated to summarize patient demographics, exposure information (activity interference) and outcome (for HADS and Improved HAQ). Normality for each continuous variable was investigated using the Shapiro–Wilk test alongside checking the distribution visually using histograms. Means (s.d.) were calculated for normally distributed data with median and interquartile range (IQR) being calculated for all non-normal data.

Correlation coefficient matrixes were calculated using Pearson’s correlation coefficient to validate whether the three outcome questionnaires, normally distributed, were associated with one another. No questionnaire resulted in a high correlation with another (a result of 0.70 or higher) and this allowed the use of all three to be included in further analysis. Each matrix resulted in eight categories showing strong correlation with at least one other category. Cronbach’s α-score of 0.99 indicated the internal reliability of these outcome questionnaires.

Simple linear regression was estimated for the association between the three outcome measures, HADS Anxiety score, HADS Depression score and the Improved HAQ total score, and the exposure—all activity interference parameters. Primarily, it was checked that the models met all assumptions of linear regression: linear association between outcome and exposure, residuals are normally distributed with a mean of zero, the residuals are independent, and there is no heteroskedasticity present. Where the models did not meet these assumptions, data were transformed and independence of residuals checked; where there was significant heteroskedasticity, robust standard errors are used (this was not required). Linear regression analysis revealed collinearity between the exposure categories, which limited analysis to only one exposure and one outcome at a time. Multivariable linear regression was used to adjust for potential confounding variables: patient age and length of disease. Four separate models were developed for potential confounding variables: model 1: unadjusted; model 2: adjusted for disease length; model 3: adjusted for patient age; and model 4: adjusted for disease length and patient age.

Interaction tests were used to investigate if the associations between exposure categories and outcome results differed due to length of disease. HADS Anxiety was shown to be the only outcome to have a significant interaction with length of disease in three activity interference questionnaires, fatigue, mood and dryness. For associations with significant interactions, length of disease was converted to a binary variable to test if a particular length of disease (in years) caused the significant finding. Due to participants having a median value of 8 years for length of disease, the continuous variable was converted to binary with 1 = length of disease 9 years or longer and 0 = all other years. All analysis was performed using the statistical software package Stata/SE 16.1 (StatCorp LLC, College Station, TX, USA).

## Results

Table 1 shows the patient demographics of those who participated in this study. Analysis found 88.59% of participants identified as female. With a median age of 63.80 years and range of 55.97 years (minimum age of 32 and maximum age of 88), most participants (50.67%) were retired at the time of study in 2014 and 66.67% had no dependents living at home. A significant portion of participants did not receive disability living allowance/personal independence payments or independent living fund, 65.33% and 76.97%, respectively.

**Table 1:** Primary Sjögren’s syndrome patient demographics

Variable	Value, *n* (%) (*n* = 149)
Gender	
Female	132 (88.59)
Male	17 (11.41)
Employment status	
Unemployed	7 (4.70)
Part time	19 (12.75)
Full time	15 (10.07)
Full time education	1 (0.67)
Housewife/husband	5 (3.36)
Retired	76 (50.02)
Unanswered	26 (17.45)
Other adults in household	
Yes	92 (61.75)
No	32 (21.48)
Unanswered	25 (16.78)
Dependents	
No dependents	100 (67.11)
Dependents in home part time	19 (12.75)
Dependents in home full time	4 (2.68)
Unanswered	26 (17.45)
Paid for non-NHS services	
Yes	28 (18.79)
No	96 (64.43)
Unanswered	25 (16.78)
Qualifications	
No formal qualifications	27 (18.12)
GCSE/O level	32 (21.48)
A level	21 (14.09)
University degree	20 (13.42)
Postgraduate degree	20 (13.42)
Unanswered	29 (19.46)
Household income (£)	
≤15 000	28 (18.79)
>15 000–26 000	21 (14.09)
>26 000–35 000	13 (8.72)
>35 000–50 000	13 (8.72)
>50 000–70 000	8 (5.37)
≥70 000	5 (3.36)
Prefer not to say	36 (24.16)
Unanswered	25 (16.78)
Disability Living Allowance/Personal Independence Payments or Independent Living Fund	
Yes	26 (17.45)
No	98 (65.77)
Unanswered	25 (16.78)
Employment and Support Allowance or Incapacity Benefit	
Yes (1)	9 (6.04)
No (2)	115 (77.18)
Unanswered	25 (16.78)

### Activity interference assessment questionnaires

Table 2 shows descriptive analysis conducted on the five different CPEQs: pain, fatigue, mood, dryness and brain fog/mental fatigue. Mean (s.d.) is shown for normally distributed data with IQR and median shown for non-normal data. All nine categories in the Fatigue Activity Interference Questionnaire were normally distributed. ‘Physical exercise’, with a mean score of 3.49 (s.d. 1.26), is the activity most impacted by fatigue. The CPEQ concerning mood found four activity categories to be normally distributed with ‘sleeping’ being the most impacted with a median score of 3 (IQR 1, 4). The Dryness questionnaire resulted in ‘sleeping’ with a mean score of 2.74 (s.d. 1.40) and ‘sexual relations’ with a mean score or 2.63 (s.d. 1.56) being the top two activities impacted. ‘Mental efficacy’ is impacted the greatest due to brain fog/mental fatigue with a mean score of 2.67 (s.d. 1.28).

**Table 2:** Activity interference (CPEQ) questionnaire results for the domains pain, fatigue, mood, dryness and brain fog/mental activity

Activity interference domain	Going to work	Performing household chores	Gardening or shopping	Socializing with others	Recreation/hobbies	Sexual relations	Physical exercise	Sleeping	Mental efficacy
Pain									
Mean (s.d.)	—	2.72 (1.22)	2.85 (1.25)	—	2.61 (1.27)	2.61 (1.56)	3.12 (1.35)	2.95 (1.36)	—
Median (IQR)	2 (1, 3)	—	—	2 (1, 3)	—	—	—	—	2 (1, 3)
Fatigue									
Mean (s.d.)	2.73 (1.37)	3.14 (1.18)	3.18 (1.20)	2.62 (1.24)	2.88 (1.20)	3.00 (1.52)	3.49 (1.26)	2.98 (1.35)	2.69 (1.17)
Median (IQR)	—	—	—	—	—	—	—	—	—
Mood									
Mean (s.d.)	—	2.32 (1.16)	2.32 (1.22)	—	—	2.53 (1.48)	2.73 (1.35)	—	—
Median (IQR)	2 (1, 3)	—	—	2 (1, 3)	2 (1, 3)	—	—	3 (1, 4)	2 (1, 3)
Dryness									
Mean (s.d.)	—	—	—	—	—	2.63 (1.56)	2.60 (1.38)	2.74 (1.40)	—
Median (IQR)	2 (1, 3)	2 (1, 3)	2 (1, 3)	2 (1, 3)	2 (1, 3)	—	—	—	2 (1, 3)
Brain fog/mental fatigue									
Mean (s.d.)	—	—	2.48 (1.24)	—	2.52 (1.20)	—	2.59 (1.33)	—	2.67 (1.28)
Median (IQR)	2 (1, 3)	2 (1, 3)	—	2 (1, 3)	—	2 (1, 3)	—	2 (1, 3)	—

CPEQ: Comprehensive Pain Evaluation Questionnaire; IQR: interquartile range.

### Hospital Anxiety and Depression Scale/Improved Health Assessment Questionnaire disability index

Descriptive statistics were performed on the three outcome questionnaires. Patients in this study scored in the ‘normal’ range for anxiety with a median score of 7 (IQR 4, 11) and depression with a median score of 5.50 (IQR 3, 8). However, further analysis shows only 53.38% of the participants scored in the normal range for anxiety while 46.62% scored either ‘borderline abnormal’ or ‘abnormal’ . The Improved HAQ total score had a normal distribution with a median value of 15.63 (IQR 37, 50).

### Regression analysis

Due to an overall strong collinearity between the activity variables, simple linear regression was performed between each CPEQ category and the three outcome questionnaires (Tables 3–5). A trend can be seen that for every point increase in a CPEQ activity score, HADS Anxiety, HADS Depression and Improved HAQ total score will also increase indicating increased levels of anxiety/depression and reduced overall quality of life. The effect size is similar between HADS Anxiety (1.25; 95% CI: 0.57, 1.93) and HADS Depression (1.12; 95% CI: 0.53, 1.71) while a larger impact is seen in the Improved HAQ (6.74; 95% CI: 2.04, 11.44). In unadjusted models, positive, significant associations were observed for all categories except ‘sexual relations’ in relation to pain interference and dryness interference for the HADS Anxiety scale, and for dryness interference for the HADS Depression scale where there was no significant association. In multivariable linear regression adjusted for length of disease, effect sizes decreased suggesting evidence of confounding. Age was likewise adjusted and not found to be a confounder.

**Table 3:** Linear regression analysis of HADS Anxiety

	HADS Anxiety		
Activity interference variable	Unadjusted β-coefficient (95% CI), *P*-value	β-coefficient adjusted for length of disease (95% CI), *P*-value	β-coefficient adjusted for age (95% CI), *P*-value
Pain interference			
Going to work	1.25 (0.57, 1.93), *P* < 0.001	0.80 (0.01, 1.49), *P* = 0.03	1.18 (0.48, 1.89), *P* = 0.001
Performing household chores	1.17 (0.56, 1.78), *P* < 0.001	0.83 (0.19, 1.46), *P* = 0.01	1.14 (0.53, 1.75), *P* < 0.001
Gardening or shopping	1.28 (0.70, 1.87), *P* < 0.001	0.97 (0.35, 1.59), *P* = 0.002	1.27 (0.69, 1.85), *P* < 0.001
Socializing with others	1.44 (0.87, 2.01), *P* < 0.001	1.17 (0.57, 1.78), *P* < 0.001	1.40 (0.82, 1.98), *P* < 0.001
Recreation/hobbies	1.41 (0.85, 1.97), *P* < 0.001	1.15 (0.56, 1.73), *P* < 0.001	1.38 (0.81, 1.94), *P* < 0.001
Sexual relations	0.13 (–0.43, 0.67), *P* = 0.66	0.02 (–0.55, 0.58), *P* = 0.96	0.09 (–0.47, 0.65), *P* = 0.75
Physical exercise	1.01 (0.46, 1.56), *P* < 0.001	0.84 (0.27, 1.41), *P* = 0.004	0.98 (0.43, 1.53), *P* = 0.001
Sleeping	1.32 (0.75, 1.90), *P* < 0.001	1.05 (0.49, 1.61), *P* < 0.001	1.31 (0.79, 1.83), *P* < 0.001
Mental efficacy	1.49 (0.88, 2.11), *P* < 0.001	1.19 (0.60, 1.78), *P* < 0.001	1.45 (0.85, 2.05), *P* < 0.001
Fatigue interference			
Going to work	1.38 (0.75, 2.01), *P* < 0.001	0.85 (0.15, 1.55), *P* = 0.02	1.28 (0.64, 1.92), *P* < 0.001
Performing household chores	1.75 (1.14, 2.36), *P* < 0.001	1.22 (0.57, 1.87), *P* < 0.001	1.71 (1.13, 2.30), *P* < 0.001
Gardening or shopping	1.79 (1.21, 2.37), *P* < 0.001	1.45 (0.82, 2.09), *P* < 0.001	1.77 (1.20, 2.35), *P* < 0.001
Socializing with others	1.80 (1.22, 2.38), *P* < 0.001	1.47 (0.86, 2.08), *P* < 0.001	1.77 (1.21, 2.33), *P* < 0.001
Recreation/hobbies	1.97 (1.42, 2.53), *P* < 0.001	1.63 (1.00, 2.25), *P* < 0.001	1.94 (1.38, 2.50), *P* < 0.001
Sexual relations	0.55 (–0.43, 1.14), *P* = 0.07	0.34 (–0.28, 0.96), *P* = 0.27	0.54 (–0.04, 1.13), *P* = 0.07
Physical exercise	1.40 (0.80, 2.00), *P* < 0.001	1.11 (0.51, 1.72), *P* < 0.001	1.36 (0.79, 1.94), *P* < 0.001
Sleeping	1.62 (1.07, 2.17), *P* < 0.001	1.36 (0.78, 1.93), *P* < 0.001	1.62 (1.12, 2.12), *P* < 0.001
Mental efficacy	1.79 (1.21, 2.37), *P* < 0.001	1.66 (1.03, 2.30), *P* < 0.001	1.75 (1.15, 2.35), *P* < 0.001
Mood interference			
Going to work	2.02 (1.26, 2.79), *P* < 0.001	1.52 (0.75, 2.28), *P* < 0.001	1.94 (1.27, 2.61), *P* < 0.001
Performing household chores	2.53 (1.99, 3.06), *P* < 0.001	2.23 (1.63, 2.82), *P* < 0.001	2.49 (1.98, 3.01), *P* < 0.001
Gardening or shopping	2.22 (1.58, 2.85), *P* < 0.001	1.82 (1.22, 2.42), *P* < 0.001	2.23 (1.72, 2.73), *P* < 0.001
Socializing with others	2.83 (2.38, 3.28), *P* < 0.001	2.60 (2.07, 3.13), *P* < 0.001	2.82 (2.36, 3.28), *P* < 0.001
Recreation/hobbies	2.45 (1.95, 2.96), *P* < 0.001	2.29 (1.71, 2.87), *P* < 0.001	2.42 (1.92, 2.93), *P* < 0.001
Sexual relations	1.19 (0.62, 1.77), *P* < 0.001	1.02 (0.41, 1.64), *P* = 0.001	1.17 (0.59, 1.75), *P* < 0.001
Physical exercise	1.80 (1.27, 2.32), *P* < 0.001	1.53 (1.00, 2.06), *P* < 0.001	1.78 (1.30, 2.27), *P* < 0.001
Sleeping	1.96 (1.51, 2.40), *P* < 0.001	1.79 (1.30, 2.27), *P* < 0.001	1.93 (1.49, 2.37), *P* < 0.001
Mental efficacy	2.33 (1.80, 2.87), *P* < 0.001	2.12 (1.55, 2.68), *P* < 0.001	2.33 (1.80, 2.86), *P* < 0.001
Dryness interference			
Going to work	1.14 (0.50, 1.77), *P* = 0.001	0.96 (0.34, 1.57), *P* = 0.003	1.04 (0.39, 1.70), *P* = 0.002
Performing household chores	0.60 (–0.02, 1.21), *P* = 0.06	0.48 (–0.15, 1.10), *P* = 0.13	0.60 (–0.01, 1.21), *P* = 0.05
Gardening or shopping	0.54 (–0.06, 1.13), *P* = 0.08	0.39 (–0.22, 1.01), *P* = 0.21	0.59 (–0.001, 1.17), *P* = 0.05
Socializing with others	0.75 (0.15, 1.35), *P* = 0.02	0.66 (0.05, 1.28), *P* = 0.03	0.73 (0.13, 1.33), *P* = 0.02
Recreation/hobbies	0.81 (0.22, 1.41), *P* = 0.008	0.63 (0.02, 1.25), *P* = 0.04	0.80 (0.21, 1.40), *P* = 0.008
Sexual relations	–0.001 (–0.57, 0.57), *P* = 1.00	0.03 (–0.54, 0.61), *P* = 0.91	–0.01 (–0.58, 0.56), *P* = 0.97
Physical exercise	0.59 (0.05, 1.14), *P* = 0.03	0.52 (–0.05, 1.09), *P* = 0.08	0.63 (0.09, 1.18), *P* = 0.02
Sleeping	0.82 (0.29, 1.35), *P* = 0.003	0.83 (0.28, 1.38), *P* = 0.003	0.79 (0.25, 1.32), *P* = 0.004
Mental efficacy	1.83 (1.23, 2.43), *P* < 0.001	1.72 (1.12, 2.33), *P* < 0.001	1.78 (1.17, 2.39), *P* < 0.001
Brain fog/mental fatigue interference			
Going to work	1.77 (1.09, 2.45), *P* < 0.001	1.28 (0.60, 1.96), *P* < 0.001	1.76 (1.08, 2.44), *P* < 0.001
Performing household chores	1.73 (1.04, 2.42), *P* < 0.001	1.13 (0.43, 1.84), *P* = 0.002	1.69 (1.07, 2.31), *P* < 0.001
Gardening or shopping	1.77 (1.23, 2.32), *P* < 0.001	1.31 (0.69, 1.94), *P* < 0.001	1.75 (1.21, 2.29), *P* < 0.001
Socializing with others	1.70 (1.14, 2.26), *P* < 0.001	1.26 (0.64, 1.88), *P* < 0.001	1.66 (1.10, 2.23), *P* < 0.001
Recreation/hobbies	1.88 (1.32, 2.44), *P* < 0.001	1.43 (0.79, 2.07), *P* < 0.001	1.84 (1.28, 2.41), *P* < 0.001
Sexual relations	0.91 (0.30, 1.52), *P* = 0.004	0.46 (–0.19, 1.10), *P* = 0.16	0.90 (0.28, 1.51), *P* = 0.004
Physical exercise	1.46 (0.94, 1.98), *P* < 0.001	1.09 (0.51, 1.67), *P* < 0.001	1.45 (0.93, 1.97), *P* < 0.001
Sleeping	1.54 (1.01, 2.07), *P* < 0.001	1.33 (0.74, 1.92), *P* < 0.001	1.51 (0.98, 2.04), *P* < 0.001
Mental efficacy	1.70 (1.17, 2.24), *P* < 0.001	1.50 (0.92, 2.07), *P* < 0.001	1.67 (1.12, 2.22), *P* < 0.001

HADS: Hospital Anxiety and Depression Scale.

**Table 4:** Linear regression analysis of HADS Depression

Activity interference variable	HADS Depression		
	Unadjusted β-coefficient (95% CI), *P*-value	β-coefficient adjusted for length of disease (95% CI), *P*-value	β-coefficient adjusted for age (95% CI), *P*-value
Pain interference			
Going to work	1.12 (0.53, 1.71), *P* < 0.001	0.76 (0.18, 1.35), *P* = 0.01	1.07 (0.47, 1.68), *P* = 0.001
Performing household chores	1.29 (0.80, 1.78), *P* < 0.001	1.15 (0.65, 1.65), *P* < 0.001	1.27 (0.78, 1.76), *P* < 0.001
Gardening or shopping	1.31 (0.85, 1.78), *P* < 0.001	1.27 (0.78, 1.76), *P* < 0.001	1.30 (0.84, 1.77), *P* < 0.001
Socializing with others	1.38 (0.94, 1.83), *P* < 0.001	1.31 (0.84, 1.77), *P* < 0.001	1.35 (0.90, 1.81), *P* < 0.001
Recreation/hobbies	1.29 (0.78, 1.80), *P* < 0.001	1.23 (0.77, 1.69), *P* < 0.001	1.26 (0.80, 1.71), *P* < 0.001
Sexual relations	0.54 (0.07, 1.01), *P* = 0.03	0.50 (0.04, 0.95), *P* = 0.03	0.50 (0.03, 0.97), *P* = 0.04
Physical exercise	0.98 (0.53, 1.42), *P* < 0.001	0.99 (0.83, 1.45), *P* < 0.001	0.95 (0.50, 1.40), *P* < 0.001
Sleeping	1.07 (0.63, 1.51), *P* < 0.001	0.83 (0.36, 1.31), *P* = 0.001	1.06 (0.62, 1.49), *P* < 0.001
Mental efficacy	1.47 (0.92, 2.01), *P* < 0.001	1.29 (0.83, 1.76), *P* < 0.001	1.45 (0.97, 1.93), *P* < 0.001
Fatigue interference			
Going to work	1.60 (1.09, 2.10), *P* < 0.001	1.28 (0.72, 1.84), *P* < 0.001	1.55 (1.04, 2.07), *P* < 0.001
Performing household chores	2.03 (1.56, 2.49), *P* < 0.001	1.78 (1.30, 2.25), *P* < 0.001	2.00 (1.57, 2.44), *P* < 0.001
Gardening or shopping	2.01 (1.56, 2.46), *P* < 0.001	1.88 (1.42, 2.33), *P* < 0.001	1.99 (1.57, 2.42), *P* < 0.001
Socializing with others	1.85 (1.40, 2.29), *P* < 0.001	1.73 (1.29, 2.18), *P* < 0.001	1.83 (1.41, 2.25), *P* < 0.001
Recreation/hobbies	2.02 (1.61, 2.43), *P* < 0.001	1.90 (1.45, 2.35), *P* < 0.001	1.99 (1.58, 2.40), *P* < 0.001
Sexual relations	0.97 (0.49, 1.45), *P* < 0.001	0.77 (0.29, 1.26), *P* = 0.002	0.96 (0.49, 1.44), *P* < 0.001
Physical exercise	1.70 (1.25, 2.15), *P* < 0.001	1.63 (1.18, 2.07), *P* < 0.001	1.67 (1.24, 2.11), *P* < 0.001
Sleeping	1.41 (1.00, 1.83), *P* < 0.001	1.19 (0.72, 1.67), *P* < 0.001	1.41 (1.00, 1.82), *P* < 0.001
Mental efficacy	1.79 (1.33, 2.25), *P* < 0.001	1.75 (1.26, 2.24), *P* < 0.001	1.77 (1.30, 2.24), *P* < 0.001
Mood interference			
Going to work	1.56 (0.96, 2.16), *P* < 0.001	1.25 (0.57, 1.94), *P* < 0.001	1.50 (0.89, 2.11), *P* < 0.001
Performing household chores	1.98 (1.53, 2.44), *P* < 0.001	1.90 (1.39, 2.40), *P* < 0.001	1.96 (1.50, 2.41), *P* < 0.001
Gardening or shopping	1.60 (1.16, 2.05), *P* < 0.001	1.43 (–0.93, 1.93), *P* < 0.001	1.61 (1.18, 2.05), *P* < 0.001
Socializing with others	2.13 (1.71, 2.54), *P* < 0.001	2.03 (1.56, 2.50), *P* < 0.001	2.11 (1.69, 2.53), *P* < 0.001
Recreation/hobbies	2.01 (1.59, 2.44), *P* < 0.001	1.94 (1.48, 2.41), *P* < 0.001	1.98 (1.56, 2.41), *P* < 0.001
Sexual relations	1.14 (0.66, 1.62), *P* < 0.001	0.92 (0.44, 1.41), *P* < 0.001	1.11 (0.64, 1.59), *P* < 0.001
Physical exercise	1.53 (1.11, 1.95), *P* < 0.001	1.45 (1.03, 1.88), *P* < 0.001	1.52 (1.13, 1.91), *P* < 0.001
Sleeping	1.38 (0.99, 1.77), *P* < 0.001	1.35 (0.93, 1.77), *P* < 0.001	1.35 (0.96, 1.74), *P* < 0.001
Mental efficacy	1.92 (1.49, 2.35), *P* < 0.001	1.84 (1.38, 2.30), *P* < 0.001	1.91 (1.47, 2.35), *P* < 0.001
Dryness interference			
Going to work	1.00 (0.45, 1.55), *P* < 0.001	0.94 (0.41, 1.47), *P* = 0.001	0.93 (0.36, 1.50), *P* = 0.002
Performing household chores	0.73 (0.21, 1.25), *P* = 0.006	0.87 (0.34, 1.39), *P* = 0.001	0.74 (0.22, 1.25), *P* = 0.005
Gardening or shopping	0.51 (–0.02, 1.00), *P* = 0.04	0.55 (0.06, 1.04), *P* = 0.03	0.55 (0.07, 1.04), *P* = 0.03
Socializing with others	0.69 (0.20, 1.18), *P* = 0.006	0.81 (0.32, 1.30), *P* = 0.001	0.67 (0.18, 1.16), *P* = 0.007
Recreation/hobbies	0.88 (0.41, 1.36), *P* < 0.001	0.92 (0.43, 1.40), *P* < 0.001	0.88 (0.40, 1.35), *P* < 0.001
Sexual relations	0.17 (–0.33, 0.66), *P* = 0.48	0.35 (–0.13, 0.82), *P* = 0.15	0.16 (–0.32, 0.64), *P* = 0.50
Physical exercise	0.36 (–0.09, 0.82), *P* = 0.11	0.48 (0.01, 0.95), *P* = 0.05	0.40 (–0.05, 0.85), *P* = 0.08
Sleeping	0.57 (0.12, 1.02), *P* = 0.01	0.69 (0.23, 1.15), *P* = 0.003	0.54 (0.09, 0.99), *P* = 0.02
Mental efficacy	1.23 (0.72, 1.74), *P* < 0.001	1.24 (0.73, 1.76), *P* < 0.001	1.19 (0.67, 1.71), *P* < 0.001
Brain fog/mental fatigue interference			
Going to work	1.52 (1.00, 2.03), *P* < 0.001	1.36 (0.80, 1.91), *P* < 0.001	1.54 (0.98, 2.09), *P* < 0.001
Performing household chores	1.64 (1.15, 2.12), *P* < 0.001	1.35 (0.80, 1.90), *P* < 0.001	1.60 (1.12, 2.09), *P* < 0.001
Gardening or shopping	1.54 (1.10, 1.98), *P* < 0.001	1.33 (0.84, 1.83), *P* < 0.001	1.52 (1.08, 1.95), *P* < 0.001
Socializing with others	1.65 (1.17, 2.12), *P* < 0.001	1.46 (0.98, 1.93), *P* < 0.001	1.62 (1.18, 2.05), *P* < 0.001
Recreation/hobbies	1.67 (1.16, 2.19), *P* < 0.001	1.53 (1.04, 2.03), *P* < 0.001	1.64 (1.20, 2.09), *P* < 0.001
Sexual relations	1.01 (0.51, 1.52), *P* < 0.001	0.65 (0.12, 1.18), P = 0.02	1.00 (0.50, 1.50), *P* < 0.001
Physical exercise	1.39 (0.98, 1.80), *P* < 0.001	1.26 (0.81, 1.71), *P* < 0.001	1.38 (0.97, 1.79), *P* < 0.001
Sleeping	1.04 (0.59, 1.49), *P* < 0.001	1.02 (0.52, 1.51), *P* < 0.001	1.01 (0.56, 1.46), *P* < 0.001
Mental efficacy	1.53 (1.11, 1.96), *P* < 0.001	1.52 (1.08, 1.96), *P* < 0.001	1.51 (1.08, 1.94), *P* < 0.001

HADS: Hospital Anxiety and Depression Scale.

**Table 5:** Linear regression analysis of Improved HAQ score

Activity interference variable	Improved HAQ score		
	Unadjusted β-coefficient (95% CI), *P*-value	β-coefficient adjusted for length of disease (95% CI), *P*-value	β-coefficient adjusted for age (95% CI), *P*-value
Pain interference			
Going to work	6.74 (2.04, 11.44), *P* = 0.005	4.35 (0.38, 8.31), *P* = 0.032	7.91 (4.05, 11.77), *P* < 0.001
Performing household chores	12.56 (9.01, 16.12), *P* < 0.001	12.15 (9.07, 15.24), *P* < 0.001	12.78 (9.81, 15.76), *P* < 0.001
Gardening or shopping	12.97 (10.10, 15.83), *P* < 0.001	12.85 (9.89, 15.82), *P* < 0.001	13.04 (10.20, 15.88), *P* < 0.001
Socializing with others	9.97 (6.57, 13.37), *P* < 0.001	9.29 (5.96, 12.63), *P* < 0.001	10.91 (7.82, 14.00), *P* < 0.001
Recreation/hobbies	10.90 (7.50, 14.29), *P* < 0.001	10.49 (7.43, 13.56), *P* < 0.001	11.28 (8.34, 14.21), *P* < 0.001
Sexual relations	5.46 (2.65, 8.26), *P* < 0.001	4.89 (2.01, 7.75), *P* = 0.001	5.76 (2.98, 8.54), *P* < 0.001
Physical exercise	8.80 (5.85, 11.75), *P* < 0.001	8.94 (5.90, 11.97), *P* < 0.001	9.09 (6.17, 12.01), *P* < 0.001
Sleeping	10.93 (8.11, 13.76), *P* < 0.001	10.08 (7.14, 13.03), *P* < 0.001	11.00 (8.31, 13.70), *P* < 0.001
Mental efficacy	9.94 (6.74, 13.13), *P* < 0.001	9.50 (5.93, 12.46), *P* < 0.001	11.39 (8.19, 1460), *P* < 0.001
Fatigue interference			
Going to work	9.84 (5.77, 13.91), *P* < 0.001	6.66 (2.94, 10.36), *P* = 0.001	10.70 (7.44, 13.95), *P* < 0.001
Performing household chores	14.77 (11.81, 17.73), *P* < 0.001	13.46 (10.22, 16.71), *P* < 0.001	15.04 (12.18, 17.90), *P* < 0.001
Gardening or shopping	14.95 (12.01, 17.88), *P* < 0.001	14.21 (11.19, 17.23), *P* < 0.001	15.05 (12.29, 17.82), *P* < 0.001
Socializing with others	11.26 (8.19, 14.33), *P* < 0.001	9.85 (6.42, 13.27), *P* < 0.001	12.25 (9.26, 15.24), *P* < 0.001
Recreation/hobbies	13.09 (10.07, 16.11), *P* < 0.001	12.14 (8.81, 15.47), *P* < 0.001	13.58 (10.66, 16.50), *P* < 0.001
Sexual relations	6.20 (3.32, 9.08), *P* < 0.001	4.50 (1.39, 7.61), *P* = 0.005	6.22 (3.34, 9.09), *P* < 0.001
Physical exercise	12.35 (9.57, 15.14), *P* < 0.001	11.45 (8.30, 14.61), *P* < 0.001	12.77 (9.86, 15.67), *P* < 0.001
Sleeping	10.78 (8.03, 13.53), *P* < 0.001	9.49 (6.33, 12.65), *P* < 0.001	10.78 (8.02, 13.54), *P* < 0.001
Mental efficacy	9.57 (6.12, 13.02), *P* < 0.001	8.70 (4.88, 12.51), *P* < 0.001	10.48 (7.02, 13.93), *P* < 0.001
Mood interference			
Going to work	6.44 (2.33, 10.54), *P* = 0.002	1.63 (–2.94, 6.20), *P* = 0.48	7.09 (2.92, 11.26), *P* = 0.001
Performing household chores	11.26 (7.96, 14.59), *P* < 0.001	9.96 (6.16, 13.76), *P* < 0.001	11.51 (8.20, 14.81), *P* < 0.001
Gardening or shopping	11.54 (8.54, 14.55), *P* < 0.001	9.89 (6.40, 13.38), *P* < 0.001	11.51 (8.51, 14.51), *P* < 0.001
Socializing with others	9.29 (5.93, 12.65), *P* < 0.001	6.25 (2.29, 10.21), *P* = 0.002	9.91 (6.54, 13.27), *P* < 0.001
Recreation/Hobbies	10.28 (7.05, 13.51), *P* < 0.001	8.95 (5.23, 12.67), *P* < 0.001	10.65 (7.43, 13.86), *P* < 0.001
Sexual relations	6.15 (3.19, 9.10), *P* < 0.001	4.06 (0.86, 7.26), *P* = 0.01	6.27 (3.32, 9.23), *P* < 0.001
Physical exercise	9.47 (6.65, 12.30), *P* < 0.001	8.16 (5.03, 11.28), *P* < 0.001	9.52 (6.71, 12.34), *P* < 0.001
Sleeping	7.92 (5.14, 10.70), *P* < 0.001	7.10 (4.00, 10.19), *P* < 0.001	8.15 (5.37, 40.92), *P* < 0.001
Mental efficacy	9.48 (6.21, 12.75), *P* < 0.001	8.08 (4.44, 11.71), *P* < 0.001	10.50 (7.20, 13.80), *P* < 0.001
Dryness interference			
Going to work	4.49 (0.31, 8.67), *P* = 0.04	3.35 (–0.09, 6.78), *P* = 0.06	5.27 (1.63, 8.91), *P* = 0.005
Performing household chores	7.28 (3.34, 11.21), *P* < 0.001	7.79 (4.26, 11.33), *P* < 0.001	7.25 (3.80, 10.70), *P* < 0.001
Gardening or shopping	6.70 (3.11, 10.29), *P* < 0.001	6.62 (3.25, 9.98), *P* < 0.001	6.55 (3.30, 9.79), *P* < 0.001
Socializing with others	6.58 (2.86, 10.30), *P* = 0.001	6.83 (3.42, 10.24), *P* < 0.001	6.73 (3.41, 10.05), *P* < 0.001
Recreation/hobbies	7.98 (4.32, 11.65), *P* < 0.001	7.53 (4.14, 10.92), *P* < 0.001	8.03 (4.81, 11.25), *P* < 0.001
Sexual relations	5.02 (2.08, 7.96), *P* = 0.001	5.36 (2.42, 8.29), *P* < 0.001	5.06 (2.12, 7.80), *P* = 0.001
Physical exercise	6.08 (2.71, 9.46), *P* < 0.001	6.23 (3.05, 9.42), *P* < 0.001	5.95 (2.92, 8.97), *P* < 0.001
Sleeping	6.44 (3.47, 9.41), *P* < 0.001	7.10 (4.01, 10.18), *P* < 0.001	6.68 (3.72, 9.64), *P* < 0.001
Mental efficacy	7.58 (3.99, 11.18), *P* < 0.001	6.86 (3.17, 10.55), *P* < 0.001	8.23 (4.64, 11.83), *P* < 0.001
Brain fog/mental fatigue interference			
Going to work	6.23 (2.47, 9.99), *P* = 0.001	1.97 (–2.07, 6.01), *P* = 0.34	8.01 (4.10, 11.93), *P* < 0.001
Performing household chores	10.67 (6.66, 14.67), *P* < 0.001	8.01 (4.01, 12.01), *P* < 0.001	11.12 (7.71, 14.52), *P* < 0.001
Gardening or shopping	10.88 (7.76, 14.00), *P* < 0.001	8.97 (5.40, 12.54), *P* < 0.001	11.06 (8.03, 14.08), *P* < 0.001
Socializing with others	9.34 (6.03, 12.65), *P* < 0.001	6.71 (3.06, 10.37), *P* < 0.001	9.79 (6.58, 12.99), *P* < 0.001
Recreation/hobbies	10.42 (7.19, 13.65), *P* < 0.001	8.51 (4.78, 12.32), *P* < 0.001	10.84 (7.63, 14.04), *P* < 0.001
Sexual relations	7.42 (4.32, 10.52), *P* < 0.001	4.61 (1.19, 8.03), *P* = 0.009	7.52 (4.43, 10.60), *P* < 0.001
Physical exercise	8.36 (5.39, 11.34), *P* < 0.001	6.30 (2.98, 9.62), *P* < 0.001	8.43 (5.47, 11.38), *P* < 0.001
Sleeping	7.26 (4.12, 10.40), *P* < 0.001	6.31 (2.77, 9.85), *P* = 0.001	7.53 (4.41, 10.66), *P* < 0.001
Mental efficacy	8.15 (4.50, 11.29), *P* < 0.001	7.14 (3.70, 10.57), *P* < 0.001	9.05 (5.90, 12.20), *P* < 0.001

Improved HAQ: Improved Health Assessment Questionnaire.

**Table 6:** Interaction analysis of the binary variable length of disease

Activity interference variable	HADS Anxiety		
	Unadjusted β-coefficient (95% CI), *P*-value	β-coefficient adjusted for length of disease (95% CI), *P*-value	β-coefficient adjusted interaction length of disease ≥9 years (95% CI), *P*-value
Fatigue interference			
Gardening or shopping	1.79 (1.21, 2.37), *P* < 0.001	1.45 (0.82, 2.09), *P* < 0.001	1.33 (0.17, 2.49), *P* = 0.03
Socializing with others	1.80 (1.22, 2.38), *P* < 0.001	1.47 (0.86, 2.08), *P* < 0.001	1.24 (0.15, 2.32), *P* = 0.03
Recreation/hobbies	1.97 (1.42, 2.53), *P* < 0.001	1.63 (1.00, 2.25), *P* < 0.001	1.40 (0.30, 2.50), *P* = 0.01
Mood interference			
Gardening or shopping	2.22 (1.58, 2.85), *P* < 0.001	1.82 (1.22, 2.42), *P* < 0.001	2.00 (0.99, 3.00), *P* < 0.001
Socializing with others	2.83 (2.38, 3.28), *P* < 0.001	2.60 (2.07, 3.13), *P* < 0.001	1.20 (0.29, 2.11), *P* = 0.01

### Interaction tests

Multiple linear regression found significant results in two questionnaires, fatigue and mood, with no apparent interaction in dryness. Within the fatigue questionnaire, three categories (‘gardening or shopping, ‘socializing’ and ‘recreation’) showed significant interaction with length of disease on HADS Anxiety (*P* = 0.03, 0.03 and 0.01, respectively). In the mood questionnaire, two categories ‘gardening or shopping’ (*P* < 0.001) and ‘socializing’ (*P* = 0.01) displayed significant interaction with length of disease on HADS Anxiety. Three categories (‘gardening or shopping’, ‘socializing’ and ‘mental efficacy’) in the dryness questionnaire were significant until length of disease was converted to a binary variable. Table 6 show that all anxiety correlation scores were significantly reduced after patients had been diagnosed with pSS for 9 years or longer.

## Discussion

Activity interference associated with fatigue was found to have the biggest impact; 60% of the highest scoring categories originated from the fatigue questionnaire with ‘physical exercise’ being the activity impacted the most followed by ‘gardening or shopping’ and ‘performing household chores’. Correlation coefficient matrixes displayed strong collinearity between the CPEQ activity categories in each questionnaire; this highlights the interwoven facet of pSS symptoms. This caused an issue with performing multiple linear regression analysis between the exposure and outcome questionnaires. If these statistical tests were conducted, no true relationship between one exposure and an outcome could have been observed. For example, if multiple regression was performed between HADS Anxiety and the mood activity assessment questionnaire, there would be no way to tell if the possible effect on the anxiety score was caused by ‘recreation/hobbies’ or ‘socializing with others’.

However, looking at the correlation between the CPEQ categories and ooutcome questionnaires does pose a vital argument as to why this research is so unique to previous studies conducted on pSS. Previous studies have conducted analysis using only one outcome measure where three were used here [2, 10–12]. By having HADS Anxiety, HADS Depression and the Improved HAQ Score, both a broader and an in-depth look into pSS patients’ lives can be achieved. Close evaluation of mental health symptoms and physical constraints is possible in this study while also allowing for an overall health assessment to be conducted. Additionally, these findings show there is no clear separation between two exposure categories. To properly understand the activity interferences experienced in pSS, future research needs to take physical and mental health into consideration.

An additional aim of this research was to view what type of relationship activity interference scores had with separate health outcome measures. Unadjusted analysis revealed that for every score increase in an exposure category (i.e. ‘going to work’) every outcome score (HADS Anxiety/Depression and Improved HAQ) would also increase. The sexual relation category seemed to have the least impact on the outcome scores. However, this could be due to low power from the questionnaire results; overall, more participants refused to answer questions concerning sexual relations compared with the other eight categories. The low response rate for the question relating to sexual relations may have introduced reporting bias if those who responded were systematically different from those who did not.

Multiple linear regression was used to adjust the data for potential confounding variables, age and length of disease. This indicated that length of disease was the main confounder in this analysis and had a positive impact (lower scores) on the three health outcomes. After adjusting HADS Anxiety, HADS Depression and Improved HAQ, length of disease resulted in a lower effect size than the unadjusted regressions. To our knowledge this is the first quantitative pSS study that adjusted for length of disease. This analysis indicates that the longer a patient with pSS is diagnosed, the better able they are to adapt their expectations and lifestyle. A qualitative study found similar results where patients learned how to adapt their lifestyle around pSS and the various symptoms [14]. By modifying their diet, types of physical exercise, or altering medications, patients were able to modify their lives and create a ‘new normal’ [14]. This trend is seen in other chronic illnesses like RA [24] and Crohn’s disease [25] where patients feel their condition, over time, is less of a burden [14, 24, 25]. This study gives additional insight into which factors limit activity the most. By tailoring treatment to these key symptoms earlier, patients may learn to manage their condition sooner after diagnosis.

### Strengths and limitations

This study used an existing dataset and is therefore limited in terms of data availability. However, missing data were minimal (*n* = 4 or 2.61% of total data) and missing completely at random, making this unlikely to influence the final analysis. The total number of participants (*n* = 149) strengthens the findings of this research. Previous quantitative studies have ranged from 42 to 185 pSS patients, making this study one of the more robust representations of pSS in the UK [2, 10–12]. As this study used an existing dataset, analysis was limited to the data available. An additional limitation to take into consideration is the measurement of the exposure variables; exposure questionnaires (mood, fatigue, dryness, and brain fog/mental fatigue) were adapted from the original CPEQ and not validated before distribution to participants. This was done as no other questionnaire existed that could measure the other potential interferences. More work is needed to validate these questionnaires to check that they measure what they are intended to. However, this study sets the scene for these questionnaires to be used for further research in a larger sample size of patients.

Another point of limitation was produced by conducting a cross-sectional study. While clear exposure and outcome variables were chosen, no temporal relationship can be determined. For example, this research cannot say if the scores associated with the Dryness Activity Interference Questionnaire caused the results in the HADS Depression score or vice versa. Still, a cross-sectional study was the only option for this research project due to the data available. Using the UKPSSR [16, 18], a snapshot of the pSS population in the UK could be obtained inexpensively in a short time frame. This allowed for analysis to be conducted 6 years after the initial questionnaires were administered without the need for participants to be contacted again.

## Conclusion

This cross-sectional study investigating which pSS symptoms have the biggest impact on daily activities using the adapted CPEQ measures found that fatigue had the biggest impact on seven activity domains: physical exercise (mean score of 3.49 out of 5 [s.d. 1.26]), performing household chores (mean 3.14 [s.d. 1.18]), gardening or shopping (mean 3.18 [s.d. 1.20]), socializing with others (mean 2.62 [s.d. 1.24]), recreation/hobbies (mean 2.88 [s.d. 1.20]), sexual relations (mean 3.00 [s.d. 1.52]) and mental efficacy (mean 2.69 [s.d. 1.17]). Additionally, this research looked at the relationship between activity interference and three separate outcome measures: HADS Anxiety, HADS Depression and Improved HAQ score. A positive association is seen between each exposure category and outcome results. As an activity interference score increases, i.e. the patient experiencing more difficulty, an outcome score will also increase. However, length of disease has been shown to lessen the overall impact of this association.

Importantly, this study looked at the association between activity interferences and mental/physical outcomes, an analysis that has not been previously explored. Results found have given further insight into how pSS affects daily life to provide additional treatment options to patients. This study shows the importance of early intervention with support for symptom management, particularly fatigue. However, this research has also highlighted the interconnectedness of symptoms and how they all impact on daily activity. One possible approach within a self-management support package is to identify and use techniques that target several symptoms at once, e.g. activity pacing techniques can be used to manage both fatigue and pain. In addition, the availability of self-management support for fatigue and associated symptoms, may result in an improvement in more than one symptom, reduce activity interference and ultimately increase quality of life. 

*Funding*: This work was funded by Arthritis Research UK (Grant 20169 and Grant 22026).

*Disclosure* *statement*: The authors have declared no conflicts of interest.

## Data Availability

The data underlying this article will be shared on reasonable request to the corresponding author.
